# Loading of a porous rock with constant micro-seismic event rate suppresses seismicity and promotes subcritical failure

**DOI:** 10.1038/s41598-025-03105-5

**Published:** 2025-06-03

**Authors:** Maria-Daphne Mangriotis, Alexis Cartwright-Taylor, Ian G. Main, Andrew Curtis, Andrew F. Bell, Ian B. Butler, Florian Fusseis

**Affiliations:** 1 School of GeoSciences, University of Edinburgh, Edinburgh, UK; 2 National Oceanography Centre, Southampton, UK; 3 Heriot-Watt University, Edinburgh, UK; 4 Advanced Structural Geology, RWTH-Aachen University, Aachen, Germany

**Keywords:** Natural hazards, Seismology

## Abstract

Catastrophic failure is the end result of progressive localisation of damage creating brittle failure on a variety of system scales in the Earth. However, the factors controlling this evolution, and the relationship between deformation and the resulting earthquake hazard, are not well constrained. Here we address the question of how to adapt operational controls in a strain-inducing laboratory experiment so as to minimize associated microseismicity. We simultaneously image the induced damage using x-rays at a synchrotron, and detect acoustic emissions which can be fed back to change operational controls on the experiment. We confirm that using continuous servo-control based on acoustic emission event rate not only slows down deformation compared to standard constant strain rate loading, but also suppresses events of all sizes, including extreme events. We develop a new model that explains this observation, based on the observed evolution of microstructural damage and the fracture mechanics of subcritical crack growth. The model is independently consistent with the observed stress history and acoustic emission statistics. Our results imply that including seismic event rate control may improve risk management of induced seismicity over a range of event magnitudes, if similar processes are relevant at larger scales.

**Supplementary Information:**

The online version contains supplementary material available at 10.1038/s41598-025-03105-5.

## Introduction

Catastrophic failure of materials occurs over variety of scales and environments, from natural systems such as landslides, volcanic eruptions and earthquakes, to engineered subsurface geoenergy systems for the transition to net-zero carbon, or with corroding or incrementally stressed infrastructure such as buildings, bridges and dams. During brittle deformation, at least part of the strain energy is released seismically, leading to potential seismic hazard. Managing the associated risk involves solving many open questions on the expected frequency-magnitude distributions, the spatio-temporal occurrences of seismicity, and the associated uncertainties. In addition to natural seismicity, earthquakes can be induced by anthropogenic activities, as a result of surface operations (adding or removing mass), extraction from the subsurface (e.g. groundwater extraction, mining for critical minerals, hydrocarbon or geothermal energy production), or injection into the subsurface (e.g. wastewater disposal, hydro-/thermo-fracturing, subsurface storage) even in areas with previously low levels of seismicity. Induced seismicity may involve relatively large magnitudes^1^, even leading to significant damage to infrastructure and in some cases loss of life. But, the anthropogenic activity causing it may itself involve only small changes in stress, due to the crust being critically pre-stressed^2^.

The triggering mechanism for induced seismicity is largely case-dependent and can involve one or more of the following^3^: (a) pore-fluid diffusion, (b) poroelastic stress transmission and aseismic slip (especially in areas beyond the influence of pore-fluid diffusion), (c) elastic stress transfer from earlier seismicity leading to triggered seismicity sequences (e.g^4^). Depending on the engineering application, the pressure, injection rate, and total injected fluid volume can vary, which can impact the spatio-temporal characteristics of induced seismicity sequences^5^ For example, carbon capture and storage (CCS), wastewater disposal (WWD) and gas storage (GS), may involve gradual, long-term injection of large fluid volumes at low pressure, whereas hydraulic fracturing (HF) would involve larger pressures during injection and smaller fluid volume. Induced seismicity sequences for low-pressure long-term (LPLT) injections show limited magnitude jumps (sudden increases in event magnitudes from any preceding seismicity) and in only a few cases trailing events (continued increases in seismicity levels after the end of injection^5^. On the other hand, there are several cases of very large jumps in seismicity in Enhanced Geothermal System (EGS) applications, even after cessation of operations (e.g. South Korea Pohang event^6^, Soultz-sous Forȇts seismicity^2^).

Stakeholders commonly deploy mandated ‘traffic-light’ systems as a risk management tool in the above applications^7^. In a traffic-light system, operations may continue as planned (green), be modified (amber), or suspended (red). Commonly, pre-set magnitude thresholds are used to determine the alarm state, often suitably low to avoid public concern and discontent. However, such systems can be unreliable if reactive controls are evoked too late in the process, causing magnitudes to continue to increase even after cessation of operations, with potentially catastrophic economical and societal effects. In fact, alternatives to standard traffic light measures can be implemented to manage seismicity, for example modulated fluid injection to constrain the amount of stored elastic energy available for rupture propagation^8^ and cyclic pressurization and pulse pumping^9^.

One of the earliest risk management protocols deployed in mining operations involved an alternative approach of using the monitored frequency of events to make decisions on risk management^10–12^, after previous observations that changes in frequency of events could act as precursors to rockbursts, outbursts, and other catastrophic failure^13,14^. Monitoring of microseismic activity revealed two categories of event: one whose rate of occurrence correlated to the volume of coal extracted, and a second – arriving with a distinctly slower wave speed and lower frequency content – which was only detected prior to outbursts. By maintaining a threshold of event rate of ‘outburst-precursor’ events, and deploying pulsed infusion firing stress release techniques when the rate was exceeded (reactive control), allowed the mine to be worked for more than four years without any major outbursts occurring, and without using maximum magnitude as a control^10–12^. In the case of seismicity induced by fluid pressure change, there is also some evidence at field scale that the likelihood of extreme events can be controlled^8^ despite the time delay associated with fluid pressure diffusion away from the well-bore, and even at depths of over 6 km. Although^8^ did not intentionally manage the seismicity by maintaining constant event rates, they achieved a fairly constant event rate as a result of modulating fluid injection. These trials suggest that augmenting management systems with event rate monitoring and feedback control could be used to reduce operational risk at field scale, at least in some cases.

The stabilisation of rock failure by maintaining constant event rate itself is not new. Such feedback control was invented by^15^, and effectively used by^16^ to slow down deformation leading to failure, thereby obtaining the first in-situ ‘images’ of quasi-static fault nucleation through inversion of arrival-time data into locations of acoustic emission (AE) hypocentres. Consequently, further studies deployed this protocol to assess the spatio-temporal characteristics of AEs (e.g^17,18^). and assess velocity changes^19^ linked to deformation, or to produce a damage zone through controlled failure^20^. A similar protocol was used by^21^ to achieve suitable time resolution in the underlying deformation process to combine direct microstructural observation (through x-ray imaging and digital volume correlation) with indirect acoustic monitoring. This allowed a more complete understanding of the process of damage localisation, including aseismic processes such as grain rolling. However, while the AE feedback protocol has been shown empirically to control failure, the mechanism by which it does so has not been explained in previous work.

Here we first develop a new fracture-mechanics based model for the stabilization of failure at constant event rate based on the experimental results of^22^ who presented experimental evidence linking tensile crack growth velocity and AE rate to the stress intensity factor by a power law, called Charles’ law. From their results, it follows that if the event rate were constant, then the stress intensity factor would also be constant. This could explain why a constant AE rate would reduce the likelihood of runaway instability and large extreme events by maintaining a sub-critical stress intensity. Assuming Charles’ law also holds for the strain softening component of deformation in compressional rock failure, we add a component to account for strain hardening deformation (as in^23^), and then test this new model on previously published experimental data for constant event rate loading^21^. We then isolate the causes and effects of AE rate control using previously unpublished results from a conventional constant strain rate test as a benchmark.

Specifically, we conduct a set of two rock compression experiments on water-saturated Clashach sandstone, comparing the two loading protocols: conventional constant axial strain rate loading (test A), and constant AE event rate loading (test B). This is the first study to compare loading protocols with simultaneous in-situ monitoring of microseismic AE events (sound), using a pair of axially-located piezoelectric P-wave sensors, and observations of microstrucutural changes (‘vision’), by means of synchrotron x-ray microtomography (µCT). Based on these comparative experiments, conducted in our x-ray transparent triaxial rock deformation apparatus, we test the following hypotheses:

1. the deformation mechanisms between the two loading protocols are different, which we can test by comparing the x-ray images showing crack development.
2. The stress history for the constant AE rate control can be prescribed by the new mathematical model, where failure is stabilized in the post-peak regime by maintaining a constant, sub-critical, stress intensity or crack extension force.
3. The number of events is lower under AE feedback control and the AE $\:b$-value (the scaling parameter of the log-linear frequency-magnitude distribution) remains relatively constant through constant AE event rate control.

Hypothesis 3 would imply amplitudes of all events would be suppressed for test B, in turn reducing the seismic component of strain and the likelihood of extreme events. We find all of these hypotheses are validated by the data.

Our new theory is validated to a high degree of precision ($\:r$ = 99%) by experimental data not involved in formulating the hypothesis. These findings transform our understanding of how constant AE event rate loading can stabilize failure, and our laboratory scale and theoretical results are consistent with previous applications at field scale, where event rate monitoring and control was directly/indirectly used to successfully mitigate hazard in coal mining and water injection scenarios, respectively. Our work provides a new scientific rationale for AE event rate control reducing the likelihood of extreme events, to be explored further in comparison with, or in addition to, other adaptive management systems.

## Theory for stabilization of rock failure at constant event rate

Local strain in the post-yield region always contains a mixture of tensile and shear deformation, transitioning from being dominated by one to the other during strain hardening (tensile) and strain softening (shear) phases respectively^21^. Here we apply a boundary condition of constant event rate, equivalent to constant stress intensity and crack growth velocity in the equivalent medium, and derive a mixture model for the transition based on fracture mechanics of an effective medium and the observations of^22^. Our model for the mechanism of stabilization of runaway instability is based on the experimental observations of^22^, who described deformation under tensile loading. We apply this framework assuming the same laws hold for compressional failure, acknowledging the additional role local hardening by crack arrest plays in the early stages of compressional rock failure, as in^23^ for the case of constant stress (creep) loading. The main difference in compressional experiments is that we cannot define the problem solely in a single mode (tensile- mode I - and mode II or III shear). Instead we use a model where the stress intensity or crack extension force $\:K$ is an effective mean field parameter, a framework commonly used in damage mechanics, and which can be used to explain the evolution of the acoustic emission event rate and the Gutenberg-Richter ‘$\:b$-value’ under increasing differential stress^24^ and the emergence of power-law steady-state brittle creep under constant load^23^.

In this framework, the representative crack velocity $\:V$ of the ensemble of fractures is assumed to be related to the effective stress intensity factor $\:K$ by Charles’ law:

1 $$\:V={V}_{c}{(K/{K}_{c})}^{n},$$

where $\:{V}_{c}$ is the critical crack growth (rupture) velocity at dynamic failure, limited by inertia to be slower than the Rayleigh wave velocity on its surfaces, $\:{K}_{c}$ is a critical stress intensity factor known as the fracture toughness and the exponent $\:n>2$ is known as the stress corrosion index. Usually $\:n$>>2 for rock samples in tensile loading where $\:K={K}_{I}$^22^, so Charles’ law implies remarkably non-linear behavior. A similar relationship is found for the acoustic emission event rate $\:\dot{N}$ in tensile loading

2 $$\:\dot{N}=\dot{{N}_{c}}{(K/{K}_{c})}^{n},$$

where the exponent $\:n$ obtained by fitting (1) and (2) to experimental data are remarkably similar^22^ (Meredith & Atkinson, 1983, again with $\:K={K}_{I}$). This implies that AE event rate $\:\dot{N}$ is an indirect measure of crack growth velocity $\:V$. Again we assume this equation also holds for the mean field stress intensity.

In single mode failure, the mean field stress intensity factor is the product of the differential stress $\:\sigma\:$, the square root of a representative crack length $\:c$, and a dimensionless factor $\:Y$

3 $$\:{K}_{i}=Y\sigma\:{c}^{1/2}.$$

More generally, in a variety of loading conditions, notably for subcritical damage during compressional failure, we might expect the exponent, denoted $\:q$ here, to differ from 1/2, so that the constant $\:Y$, which also depends on the loading conditions for a finite sized sample, does have dimensions. For example, in the special case of double torsional loading, *q = 0*^22^. If the growth of a crack is inhibited by the loading, for example a tensile crack growing under a compressive stress field, then leads to crack arrest and new cracks nucleating elsewhere, producing distributed deformation in the sample. This mechanism dominates early in the loading cycle (e.g^25^. , validated by the µCT images of^21^) and results in material hardening (the differential stress continues to increase with time). There is then a transition to hybrid or shear-dominated fracture mechanisms later in the cycle, resulting in material softening and a reduction first in the rate of stress increase and then an actual reduction or material softening, often associated with localisation of deformation (see also^25^, also validated by the µCT images of^21^. The softening and hardening processes can be described most simply by generalising (3) to the form

4 $$\:K=Y\sigma\:{c}^{{q}_{s}}\:\text{a}\text{n}\text{d}\:K=Y\sigma\:{c}^{-{q}_{h}},\:$$

for hardening and softening processes, respectively^23^, where both $\:{q}_{s}$ and $\:{q}_{h}$ are positive The negative sign prior to $\:{q}_{h}$ then implies negative feedback on further crack growth by reducing the stress intensity factor as $\:c$ increases, representing the inhibition of tensile crack growth in a compressive stress field (also a component of the model of^26^, whereas the positive sign for $\:{q}_{s}$ implies positive feedback, e.g. due to localized shear on a deformation band.

^23^ assumed a linear mixture of hardening and softening processes to explain the three-stage creep law and the emergence of a power law relation between stress and strain rate under constant stress loading, without having to invoke a separate mechanism for steady-state or secondary creep. Here we change the boundary condition from constant stress to constant event rate to respect the AE loading control protocol, and assume a superposition of the two processes with a time-dependent weighting function in the linear superposition, to respect the observed transition from hardening-dominated to softening dominated fracture observed in the microstructures in^21^.

At constant event rate loading $\:\dot{N}$ is constant, and so is $\:K$ and $\:V$ from (1) and (2). In this case, stress and crack lengths are both functions of time

5 $$\:\sigma\:=\sigma\:\left(t\right)\:\:;\:\:\:\:c={c}_{0}+Vt.$$

From Eqs. (1)-(4), we find for a softening process

6 $$\:K=Y\sigma\:\left(t\right){\left({c}_{0}+Vt\:\right)}^{{q}_{s}}=A{\sigma\:\left(t\right)(1+t/{T}_{s})}^{{q}_{s}},$$

where $\:A=\:Y{{c}_{0}}^{{q}_{s}}$ is a constant and the characteristic softening time $\:{T}_{s}={c}_{0}/V$. For constant $\:K$

7 $$\:\sigma\:\left(t\right)=\left[K/\left(Y{c}_{o}^{{q}_{s}}\right)\right]{\left(1+t/{T}_{s}\right)}^{-{q}_{s}}={\sigma\:}_{0}{\left(1+t/{T}_{s}\right)}^{-{q}_{s}},$$

where $\:{\sigma\:}_{0}=\sigma\:(t=0)\:.\:\:\:$A similar equation holds for the hardening process

8 $$\:\sigma\:\left(t\right)=\left[K/\left(Y{c}_{o}^{{q}_{h}}\right)\right]{\left(1+t/{T}_{h}\right)}^{{q}_{h}}={\sigma\:}_{0}{\left(1+t/{T}_{h}\right)}^{{q}_{h}}$$

If the ratio of initial crack size to initial crack growth velocity during AE feedback control is the same for both processes, then $\:{T}_{h}={T}_{s}$. Here we allow both to be independent variables.

Equation (7) predicts $\:\sigma\:\to\:0$ as $\:t\to\:\infty\:$. In a compressional experiment it is more usual to find $\:\sigma\:\to\:{\sigma\:}_{F}$ as $\:t\to\:\infty\:$, where $\:{\sigma\:}_{F}$ is the frictional sliding stress, i.e. the steady state after a contiguous fault has developed. With this boundary condition we have

9 $$\:\sigma\:\left(t\right)={\sigma\:}_{F}+({\sigma\:}_{0}-{\sigma\:}_{F}){\left(1+t/T\right)}^{-{q}_{s}}.$$

If there is no strong asymptote within the data range, then the net stress from an evolving mixture of hardening and softening that respects the transition from one to the other using a weighting function $\:w\left(t\right)$ can then be expressed in the form

10 $$\:\sigma\:\left(t\right)={\sigma\:}_{0}\left[w\left(t\right){\left(1+t/{T}_{h}\right)}^{{q}_{h}}+{[1-w\left(t\right)]\left(1+t/{T}_{s}\right)}^{-{q}_{s}}\right]$$,

Where

11 $$\:w\left(t\right)=\text{exp}\left(-\frac{t}{{T}_{w}}\right),$$

and $\:{T}_{w}$ is a characteristic time for the transition.

## Experiment description

We used Clashach sandstone, a Permian aeolian sandstone from Morayshire, Scotland, as our experimental material. It is a highly cemented (17% porosity), well-sorted, quartz-rich arenite with > 92% quartz grains, < 8% K-feldspar and subordinate lithics with fine to medium grains 250–400 μm in diameter^27^.

The experimental setup and testing protocol are described in detail by^21^ along with a detailed description of the type of data recorded. This includes x-ray (µCT) volumes, later processed via digital volume correlation (DVC) to obtain local and overall strain estimates, continuous passive microseismic recordings from two piezoelectric P-wave sensors axially-located at the piston ends, and mechanical ram pressure (measured with an external pressure sensor calibrated for axial stress using a load cell) and LVDT axial piston displacement data (calibrated for axial sample strain by correcting for the rig stiffness).

The triaxial compression experiments were conducted in our lightweight x-ray transparent triaxial deformation apparatus, Stór Mjölnir (Figure S1), constructed of grade 5 titanium alloy, with an x-ray transparent pressure vessel made of 7068-T6 aluminium alloy, installed on the x-ray microtomography rotation stage in Experimental Hutch 1 (EH1) of beamline I12-JEEP^28^ at the Diamond Light Source. The apparatus accommodates cylindrical samples of 10 mm diameter and 25 mm length; small enough to obtain the micron-scale resolution achievable with synchrotron µCT imaging. Clashach sandstone cores were cut to size using a diamond core drill and then ground flat and parallel on a lathe. The samples were jacketed in silicone tubing and a honey-like molasses was used as a couplant between the acoustic transducers and the pistons, and between the pistons and the sample ends to provide good transmission for both shear and compressional waves over a wide range of frequencies^29^.

**Fig. 1:**
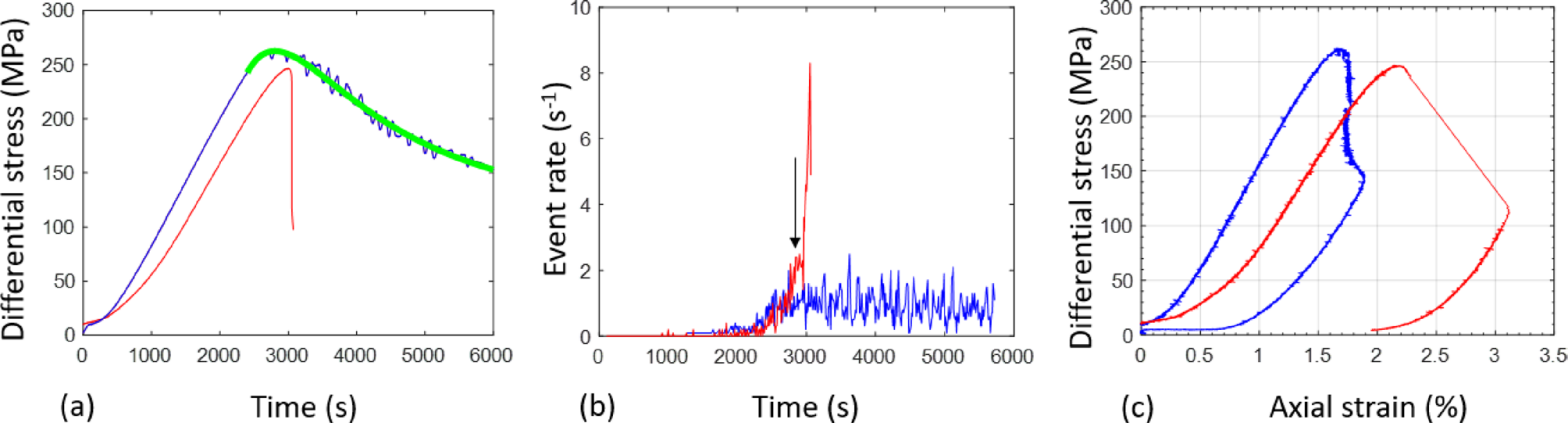
(**a**) Differential stress history, (**b**) Event rate history, (**c**) Differential stress versus axial strain for test A (red line) and test B (blue line). Green line in (**a**) shows the fitted curve using the strain hardening and softening mixture model (Equation S23) derived from Charles’ law for subcritical crack growth (Supplementary Information S3). Black arrow in (**b**) shows the time (~ 1 min duration) during which an event rate of 2 events/s was observed before rapid event rate increase immediately before peak stress.

The two experiments were performed at ambient temperature under water-saturated, drained conditions. A constant radial effective pressure ($\:{P}_{eff}$) was applied and maintained at 20 MPa throughout each test, with a confining pressure $\:{P}_{c}$ of 25 MPa and pore fluid pressure $\:{P}_{p}$ of 5 MPa, where $\:{P}_{eff}={P}_{c}-{P}_{p}$. Deionised water was used as the pressurizing fluid. A hydrostatic starting pressure condition (zero differential stress) was achieved by simultaneously increasing the axial pressure to match the effective pressure. Two tomographic reference scans were acquired prior to loading (at zero differential stress) to obtain the initial state of the sample and to characterise the error in the digital volume correlation by correlating two volumes in which the state of the sample was identical (e.g., Suppl. Figure S7 in^21^. After these initial scans, the Clashach sandstone samples were loaded continuously under a constant axial strain rate loading at 10^−5^ s^−1^, achieved by fluid delivery to the hydraulic actuator at a constant rate. We used this strain rate for three reasons: i) Ojala et al.^30^ showed that the Omori exponent for experimental foreshocks in sandstone decreases with decreasing strain rate, reflecting a steeper increase in AE event rate prior to failure, which suggests that the onset of the AE feedback control may be increasingly sensitive, and therefore less effective, with slower strain rates, ii) deformation proceeds relatively rapidly, which allows for more experiments to be conducted during our limited time at the beamline, and iii) it is commonly used for laboratory experiments so is useful for comparison with other studies. This deformation rate was maintained until failure for the constant strain rate loading test (test A), but for the constant AE event rate loading test (test B), the loading protocol switched to constant AE event rate servo-control, regulating the applied stress by slowing down the stroke rate of the ram or even allowing it to go slightly into reverse, as soon as the desired AE event rate (1 event/sec) was reached (Fig. 1). This AE event rate was established through extensive in-house testing, prior to the experimental campaign, to be the optimum event rate for effective feedback control that ensured control was early enough to prevent dynamic failure but late enough to avoid a sudden increase in strain rate and could be maintained throughout failure. The AE event rate was maintained at 1 ± 1 event/sec throughout the remainder of the test. This enabled the failure time to be extended from ~ 1 min to ~ 50 min, equivalent to an average bulk strain rate of 10^−7^ s^−1^, and sufficient to capture 18 high-quality x-ray µCT volumes after peak stress.

Our feedback control system (see Suppl. Note 1 in^21^ for more detail) drives the stepper motor of the software-controllable, ultra-high pressure Cetoni Nemesys XL syringe pump that drives the hydraulic actuator. When an AE event is detected, based on exceedance of a pre-defined amplitude threshold, the AE acquisition system outputs a pulse to our control system via an Arduino microcontroller board. Each pulse report is a pair *(N*,* dt)* where *dt* is the time since the last report and *N* is the number of pulses seen during that interval. Any report where *N* > 0 is treated as a single AE event (i.e., *N* = 1), and for each pulse report *i*, an AE event rate over the report interval is *N/dt*. A rolling estimate of pulse rate, *R*, is maintained by applying a low pass filter with a configured cut-off frequency *f*, as follows: *R*_*i*_ *=* α *R*_*i−*1_ *+*(α – 1) *N/d*, where α = *e*^*− f dt*^. This gives an AE event rate signal which peaks when an event occurs and decays exponentially at a configurable rate (key tuning parameter). An equivalent low pass filter is applied to the actuator pressure and displacement signals. Our software implements conventional PID control and the controller can operate with multiple modes, each implementing a constant set-point or a linear ramp, whereby several control modes (e.g., constant actuator pressure followed by constant strain rate) can be used to build up to a point where the AE event rate control can take over.

Operando x-ray tomographic imaging was achieved with a 53 keV monochromatic beam detected by a PCO.edge light sensor with I12 in-house optical module of 7.91 × 7.91 μm per pixel resolution. Individual scans were acquired in ~ 40 s and consisted of 1800 projections with a 0.0035 s exposure time. At each end of the sample, 0.2 mm (next to the piston) was not captured due to limits in the X-ray field of view, which had a vertical dimension of 12 mm. Tomographic volumes of the whole sample, comprising two discrete overlapping scans of the top and bottom of the sample, with vertical translation in between, were acquired every 85 s throughout the experiments. The tomographic images were reconstructed and then processed by means of digital volume correlation (DVC) between adjacent pairs of x-ray tomograms to obtain the incremental 3D strain fields using the image analysis software SPAM^31^. See^21^ for full details of tomographic reconstruction, DVC procedure, and the piezoelectric sensors and acoustic data acquisition system.

**Fig. 2:**
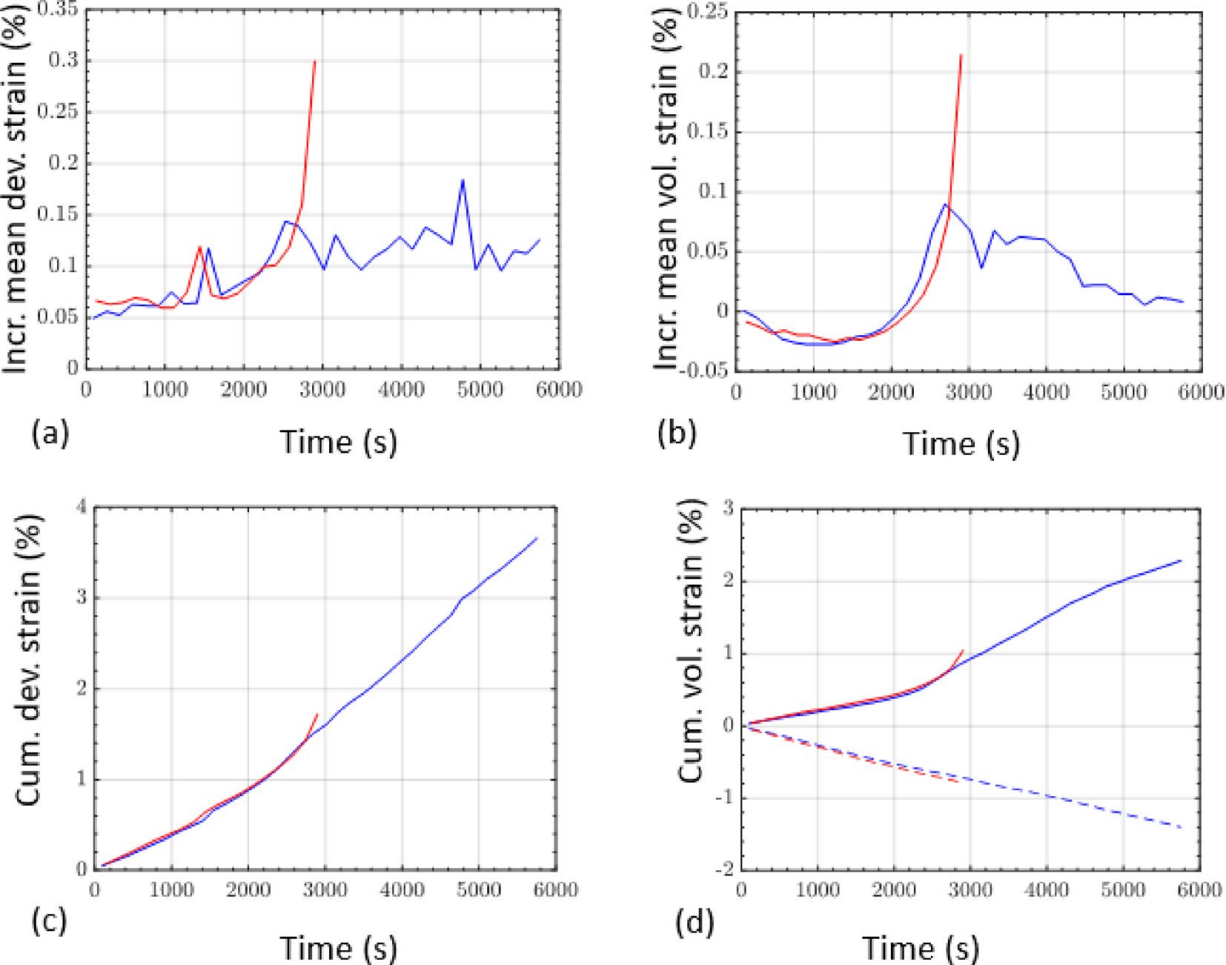
(**a**) Incremental mean deviatoric strain history, (**b**) Incremental mean volumetric strain history, (**c**) Cummulative deviatoric strain history, (**d**) Cummulative volumetric strain history for test A (red line) and test B (blue line). Negative/positive volumetric strain (bold/dashed lines) represent compaction/dilation, respectively. These plots show that average local strain evolution was similar between the two samples until the constant AE event rate loading protocol prevented critical acceleration of the average local strain rate.

**Fig. 3:**
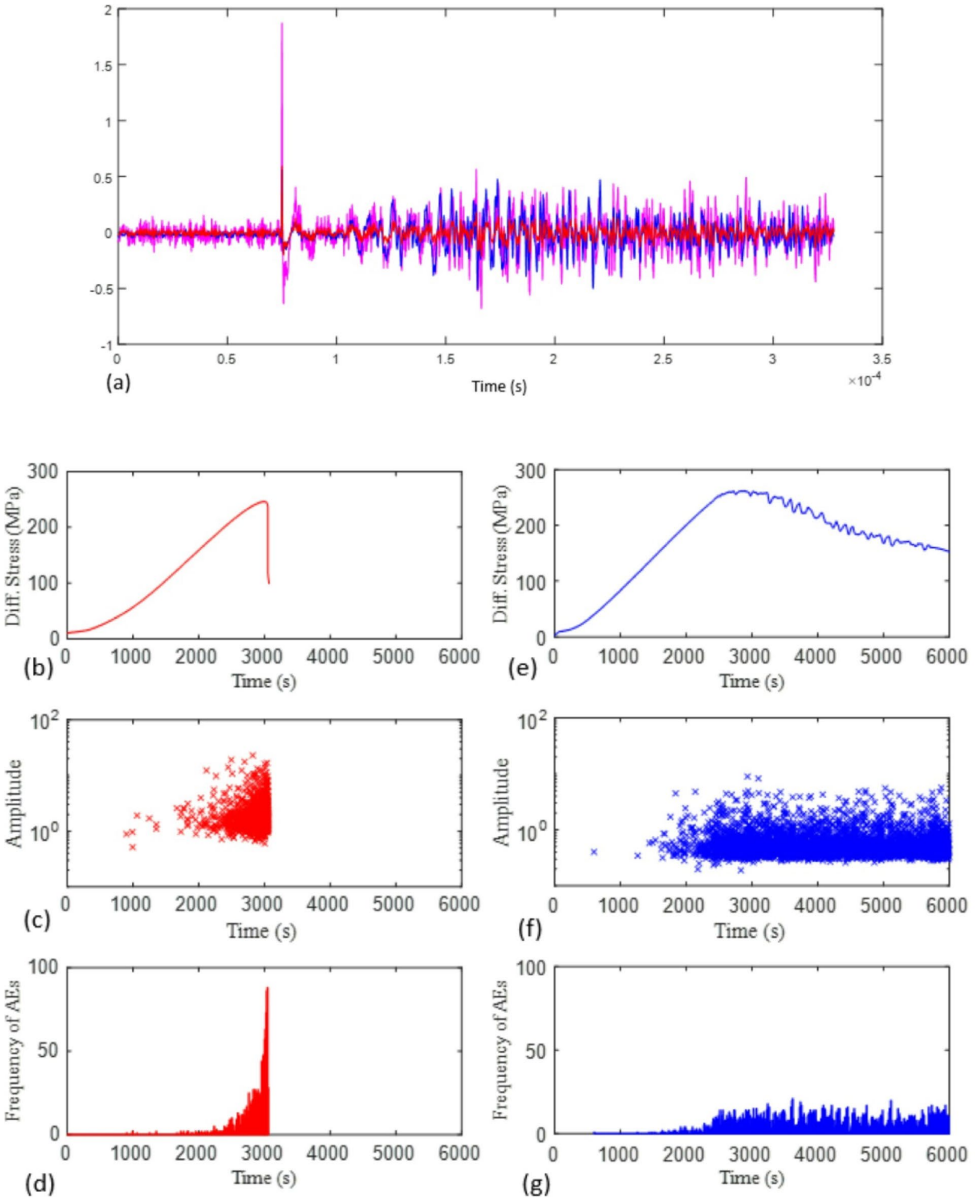
(**a**) Seismic trace of an acoustic pulse originating from the top sensor, recorded at the bottom sensor. Red and blue lines correspond to original recording from test A and B, at 60 and 70 dB respectively. Magenta line shows the recording from test A rescaled to 70 dB. Differential stress history, seismic amplitude evolution, and AE frequency evolution for test A (red) and test B (blue) are shown in (**b**–**g**), respectively.

We continuously recorded AE during loading for the two tests, at a sampling rate of 0.02 µs. Test A was recorded with a pre-amplification gain of 60 dB, based on a benchmark pencil lead-break test in our laboratory. However, the infrastructure at the synchrotron required long cables to run from the imaging hutch out into the stack of data loggers in the observation room, resulting in significant electronic noise in the seismic recordings for test A. In an effort to increase signal-to-noise ratio for the subsequent test B, we changed the gain setting to 70 dB. The absolute noise levels after calibration for this change were similar in both tests (Fig. 3), which confirmed that the noise origin was external to the loading apparatus, most likely radio frequency interference (RFI) from pump motors in the experimental hutch. After correcting the recordings for the difference in gain, we estimated the AE amplitudes from the maxima of their Hilbert envelope (Fig. 3). As a result of the lower signal-to-noise ratio recorded in test A compared to B, we could not pick as many low amplitude events in these recordings. In addition, test A involved several significantly larger events than test B due to the more dynamic loading conditions. This was associated with an acceleration in the average local incremental dilation and shear strain (Fig. 2) and occurred during the time period where loading protocol diverged between test A and B (i.e. after the AE rate feedback control kicked in). For test A, the AE event rate increased first exponentially and then as a power-law (Fig. 3)—a common characteristic when approaching catastrophic failure^32^. A total of 2235 and 3660 events were recorded for tests A and B respectively.

## Data analysis and results

### Mechanical behaviour

The stress-strain curves in Fig. 1 show the bulk mechanical behaviour of the two samples. The major differences in mechanical behaviour occur after the onset of AE control, but there are small differences in the stress-time evolution under the same loading conditions, even though the temporal evolution of the mean internal strain was similar (Fig. 3). The reason why the strain trends are similar, but the stress trends are deviating between the two tests, is due to differences in stiffness, as shown in Fig. 1c. The area under the bulk stress-strain curve indicates the mechanical work done in the samples, with constant strain rate loading conditions requiring more work (Fig. 1c). Even though samples were selected from the same block, they show natural variability to some degree, with heterogeneity statistically accentuated due to their size being small. The sample used in the constant strain rate test happened to be initially less stiff (Fig. 1c), compacting to a greater extent before entering the quasi-elastic regime. After yield, the initially softer material experienced a lower peak differential stress, even though it was loaded under more dynamic conditions post-yield.

We fit the model of Eqs. (10) and (11) to the observed stress history in Fig. 1a. If the measured time since the start of the loading phase and time of onset of AE control are respectively $\:{t}_{raw}$ and $\:{t}_{o}$, then the time in these equations is $\:t={t}_{raw}-{t}_{o}$, where $\:{t}_{o}$ is a fixed parameter. Similarly, $\:{\sigma\:}_{0}=\sigma\:\left(t=0\right)$ is also a fixed parameter that can be read from the data output at experimental time $\:{t}_{o}$. We further reduce the number of free parameters by initially assuming $\:{q}_{s}={q}_{h}=1/2$. There is no strong evidence for a finite asymptote to the stress evolution so we also initially assume $\:{\sigma\:}_{F}=0$. In this case, the free parameter vector is then ($\:{T}_{h},{T}_{s,\:}\:{T}_{w})$. While $\:{q}_{h}$ and $\:{q}_{s}$ may take on other values, and $\:{\sigma\:}_{F}>0$ in the general case, these additional free model parameters were not required here to obtain a good fit. We find that our model validates the observed differential stress evolution after the onset of AE control with a high regression coefficient (99%) yielding hardening and softening time scale parameters $\:{T}_{h}$ and $\:{T}_{s,\:}$, respectively, equal to 869.9 s and 1025 s and a transition time $\:{T}_{w}$ of 2088.

Assuming a maximum crack length of 800 μm (cmax), minimum crack length of 18 μm (cmin), as observed from the x-ray results for maximum and minimum crack sizes, and using time equal to the duration of the AE rate control, $\:{T}_{d}$=3600 s, yield an effective medium crack velocity stabilized at $\:V=\frac{{c}_{max}-{c}_{min}}{{T}_{d}}=\:$0.217$\:\:\mu\:$m/s.

### Micro-mechanics of failure

**Fig. 4:**
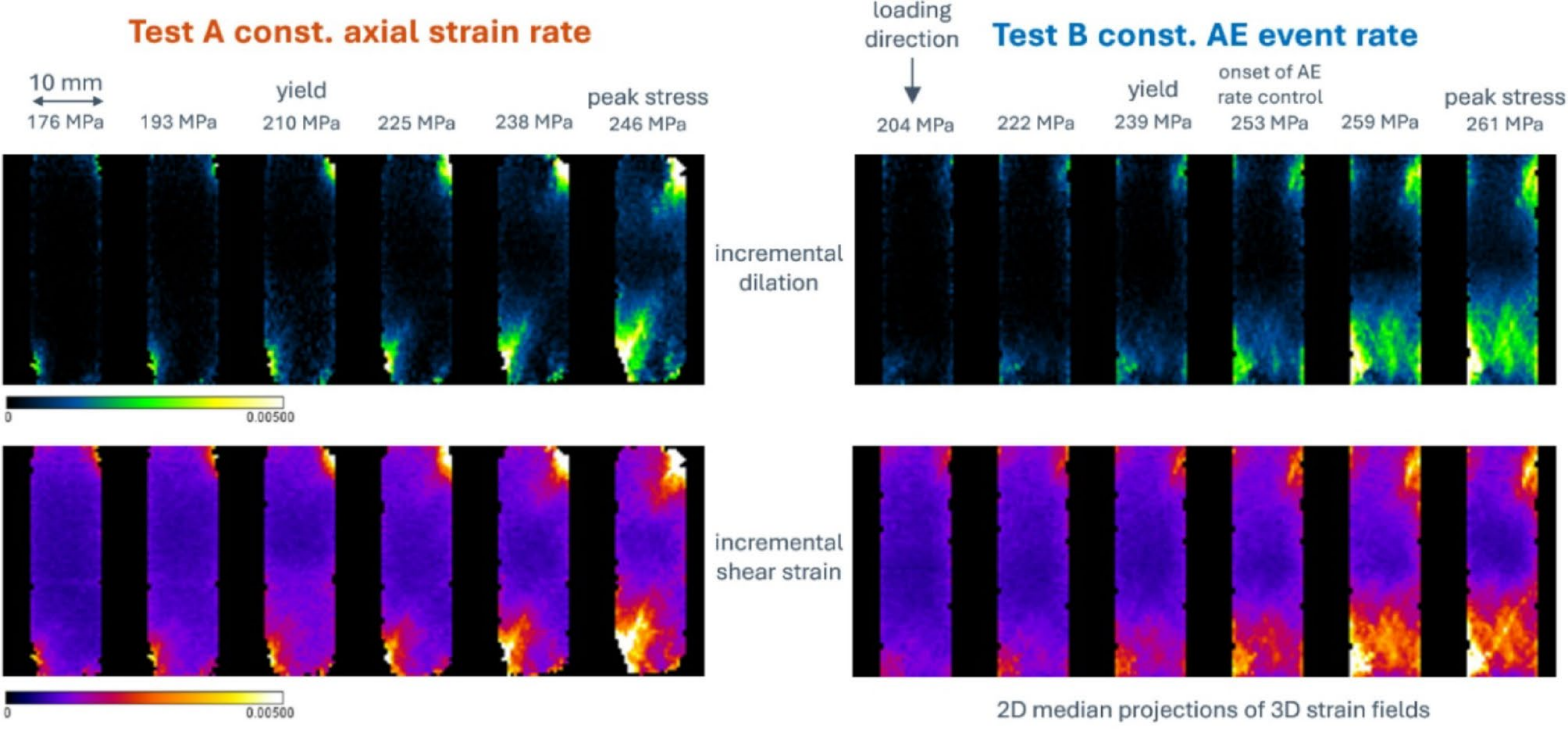
2D median projections of 3D incremental dilation and shear strain fields. Along-strike projections for sample loaded under left constant axial strain rate and right constant AE event rate from just before yield to peak stress. It is apparent that strain localisation occurs earlier under a constant strain rate and there is no evidence for the exploratory strain localisation seen in sample loaded under a constant AE event rate. Differences in differential stress values at equivalent stages of deformation relate to the difference in stiffness between the two samples. The three strain fields up to yield observed in Test B prior to the onset of AE rate control were acquired under constant strain rate loading and are therefore directly comparable with the equivalent three strain fields up to yield observed in Test A.

Differences in the micro-mechanics between the two loading protocols can only be compared between yield and peak stress, and post-failure (by comparing the x-ray images), because the constant strain rate test fails dynamically and has no data during the associated sudden stress drop. Figure 4 shows the evolution of incremental local dilation and shear strain fields, as 2D along-strike projections of the 3D strain volumes estimated from DVC between adjacent x-ray volumes, from just before yield to peak stress. Dilation and shear strain were strongly correlated in both samples and strain localised initially in the bottom left corner. However, under a constant strain rate (test A), localisation along the eventual failure plane occurred earlier, compared with the exploratory localisation seen along several potential failure planes under constant AE event rate loading (test B). Specifically, strain localisation along the failure plane in test A is observed in the local strain increment immediately before peak stress at 238 MPa (Fig. 4 left panel), with localised en-echelon tensile microcracks observed in the x-ray images at peak stress of 246 MPa, but not earlier. This relates in test B to the period of yield and strain hardening that followed the onset of AE rate control, with AE rate control beginning three local strain increments prior to peak stress in test B. Final strain localisation along the failure plane in test B occurs after exploratory strain localisation on competing planes (as seen in Fig. 4 right panel, leading up to and at peak stress) and within the strain softening region at 257 MPa just after peak stress with microcrack localisation observed slightly later at 238 MPa (as seen in^21^).

**Fig. 5:**
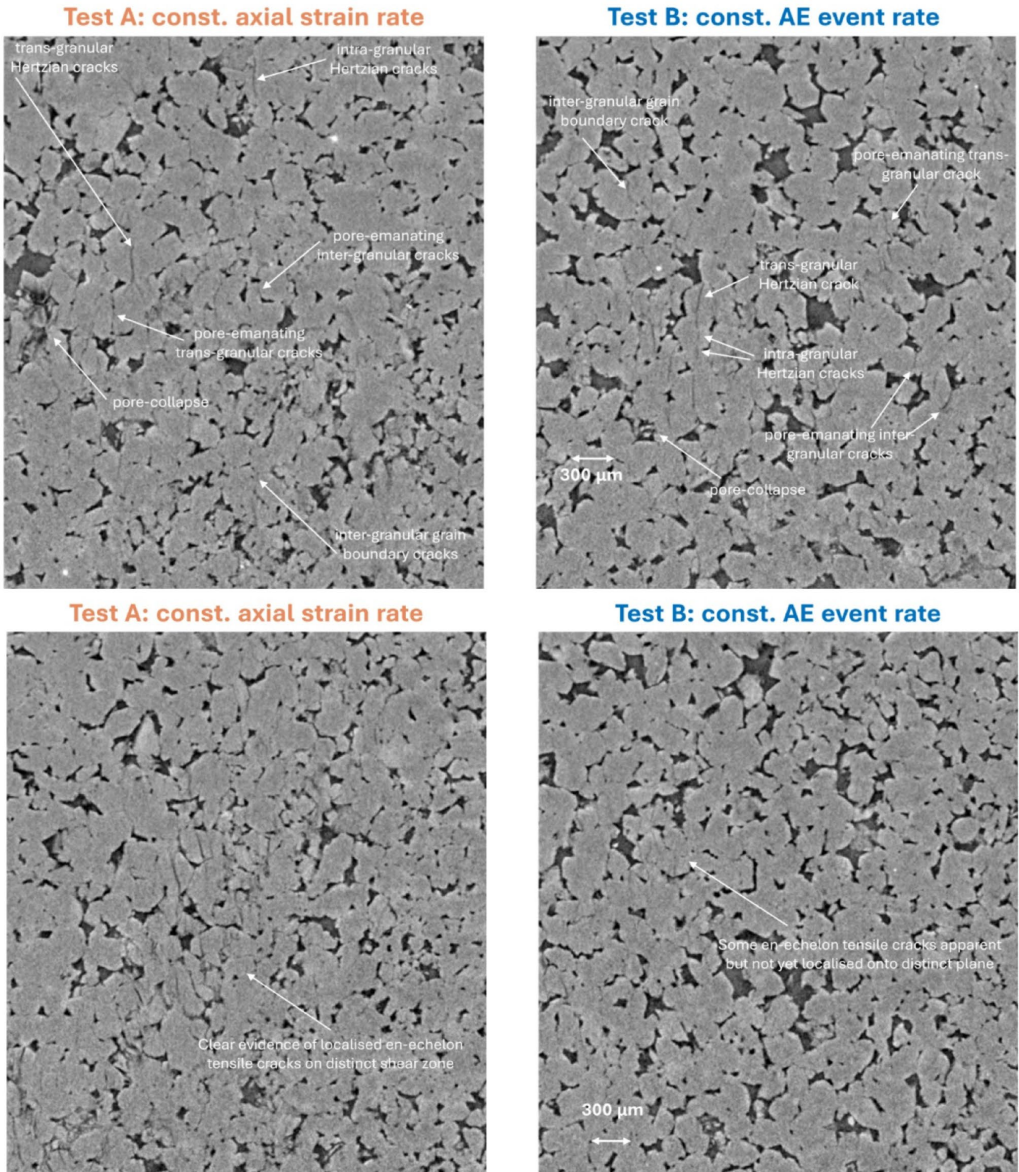
Selected 2D greyscale slices showing the microstructure in each sample at peak stress. Top: both samples show similar nucleation mechanisms of pore-emanating, Hertzian and grain boundary cracks, with similar crack lengths and apertures. Bottom: evidence for earlier localisation of en-echelon tensile cracks along an inclined plane (shear zone) under constant strain rate (left) than under constant AE event rate (right).

**Fig. 6:**
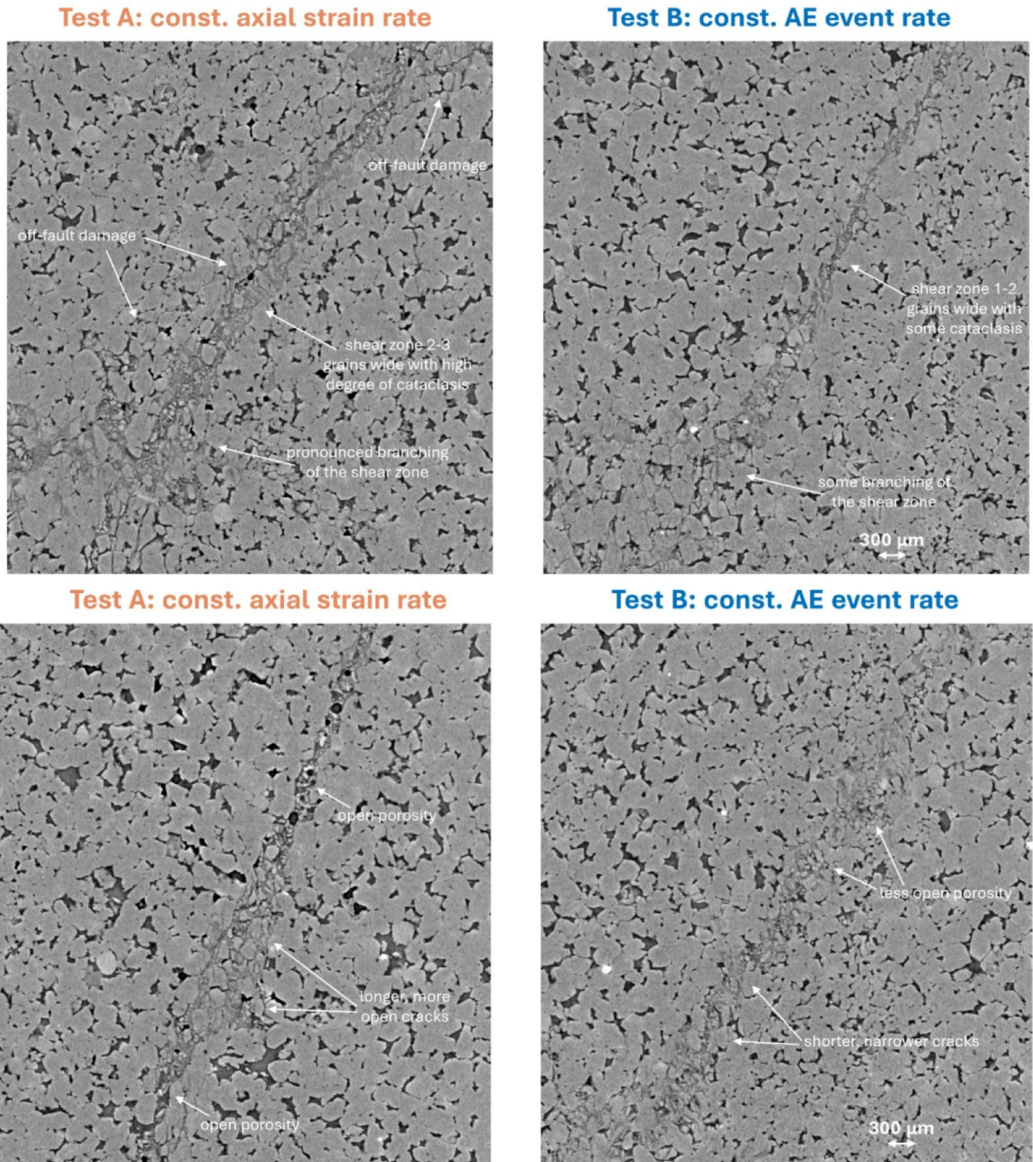
Selected 2D greyscale slices showing the microstructure in each sample post-failure after unloading. Top: evidence for broader shear zone with more cataclasis, more off-fault damage, and pronounced branching under constant strain rate (left). Bottom: evidence for more porosity in shear band, longer and more open cracks under constant strain rate (left).

Comparing 2D vertical x-ray slices from the high-strain regions in each sample at peak stress (Fig. 5), the micro-mechanics of deformation between the two experiments were broadly similar (Fig. 5 top pair of images). Microcrack damage accumulated through localised pore collapse, as pore-emanating and Hertzian tensile intra- and trans-granular cracks and as pore-emanating and grain boundary shear and tensile inter-granular cracks. These features were comparable in length and aperture, and trans-granular microcracks tended not to extend beyond two grains before deviating along grain boundaries in both cases. One important difference between the two samples was that en-echelon tensile microcracks had already localized along the eventual failure plane at peak stress in the sample deformed under a constant strain rate but not under a constant AE event rate (Fig. 5 bottom pair of images). This did not occur until in test B until the sample had experienced some strain softening after peak stress^21^. Post-failure (after unloading) the differences between the two samples were significant (Fig. 6). Microcracks were longer and more open in the test A sample than in the test B sample. The visible proportion of damaged rock was greater, with a broader shear zone around 2–3 grains wide (compared with < 1–2 grains in the sample loaded under constant AE event rate) and a greater degree of cataclasis and gouge present throughout. There was also more porosity and a greater number of intergranular cracks within the shear zone, possibly due to the larger degree of cataclasis and therefore less cohesion between grain fragments and/or more slip along the shear zone during failure due to the faster slip rate in test A. Manually measured maximum crack lengths were similar but slightly longer in the sample deformed under a constant strain rate (1000 μm for test A compared with 800 μm for test B). Fragment rotation in the shear zone was evident in both samples. Off-fault microcracking was limited, but there were some trans- and inter-granular microcracks that extended up to four to five grains long in both samples. These were more common in the test A sample and tended to be more open. Finally, branching of the fault zone appeared to be more pronounced in the test A sample, most likely as a consequence of the faster slip rate during failure in test A. The residual stress supporting frictional sliding after failure was lower in test A indicating a lower friction coefficient, likely resulting from the larger degree of cataclasis and reduced cohesion in the shear zone in this sample compared with the test B sample. Images showing the post-failure damage across the whole of each sample can be found in (Figures S2 and S3).

### Frequency-magnitude distributions

**Fig. 7:**
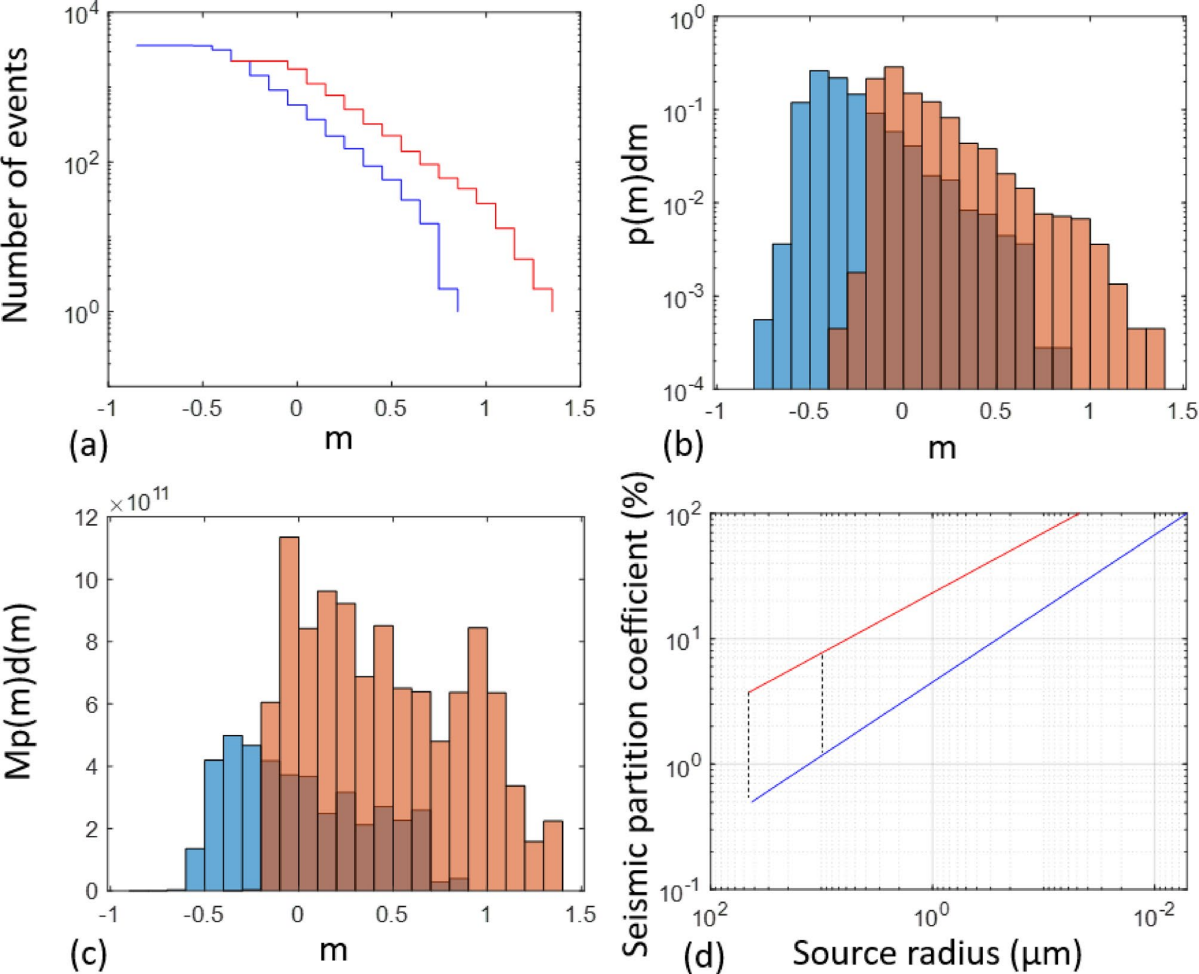
(**a**) Cumulative number of events versus seismic magnitude for tests A (red) and B (blue), (**b**) incremental probability versus seismic magnitude distribution for tests A (brown) and B (blue), (**c**) incremental moment versus seismic magnitude distribution, (**d**) seismic partition coefficient (%) versus source radius (note reverse scale, with smaller dimensions to the right; also note the figure is truncated at 100%, which is physically the upper bound for the seismic strain partition coefficient). The two vertical dotted lines show the seismic partition coefficient estimates for the extrapolated smallest crack length from our data (left line) vs. the smallest assumed crack based on the results of Alyafei et al. (2013) (right line).

The Gutenberg-Richter (G-R) law takes the form: $\:logF\left(m\right)=a-bm$, where here *F(m)* is the incremental frequency of occurrence of earthquakes with magnitudes in the range $\:m\pm\:\frac{\delta\:m}{2}$, $\:\delta\:m$ is the bin width, and *a*, *b* are constants. We estimated the slope *b*, known as the *b*-value, following the protocol of Roberts et al.^33^, who address potential bias from incorrect identification of the threshold for the completeness magnitude *m*_*C*_. The uncertainty in *b*-value (to one standard deviation) was estimated as $\:\frac{b}{\surd\:N}$, where *N* is the number of events above *m*_*C*_^34^. The estimated *b*-values for optimal *m*_*C*_ of 0.1 for test A and − 0.3 for test B are 1.86 ± 0.06 and 1.94 ± 0.04 for test A and B respectively (Fig. 7). The difference in the two best estimates with respect to their standard errors corresponds to a two-tailed P-value statistic of less than 0.0001, considered ‘statistically significant’ in the Welch unpaired test. Nevertheless, the difference in $\:b$-values is subtle and almost within the estimated errors. In both cases the relatively high *b-*value (*b* > 1.5) implies deformation is dominated by more numerous smaller events (see Supplementary Information Text S1).

Next, we explored the temporal variation of *b*-values for both tests (Supporting Information, Table S1). The *b*-value is constant within error as a function of time, based on non-overlapping (hence independent) sequences of 500 events, 2 sequences for test A and 7 sequences for test B. Due to the difference in gain between the two tests, Test A has fewer events above the magnitude of completeness, higher uncertainty in *b*-value, and fewer independent time windows to establish any temporal $\:b$-value trends.

### Seismic strain partition coefficient: estimates from observed seismicity

The quantification of aseismic slip and the associated partition of strain between seismic and aseismic processes are important open questions in seismic hazard quantification. The partition between seismic and aseismic deformation directly influences the detectability of seismic precursors to failure. For example studies of large earthquakes at plate-bounding faults show seismic foreshocks are commonly associated with areas of aseismic slip^35,36^. For most injection applications, the seismic moment released is much smaller than that estimated from injected volumes^37^, whereas for some volcanic settings there is a low ratio of cumulative seismic moment released by earthquakes through time, compared to the total expected from the total strain measured from edifice displacements^38^. This ratio is sometimes called the seismic strain partition coefficient, and can be calculated using the method outlined by^39^. Aseismic deformation has also been observed in numerous laboratory loading tests (e.g^40–42^). , and estimated to constitute approximately 99% of total deformation^43,44^.

From the frequency-magnitude distribution, we estimated the incremental probability, *p(m)dm*, and multiplied it by the seismic moment, *M(m)*, to obtain the incremental moment distribution, *M(m)p(m)dm*(Fig. 7). These weighted histograms can be used to estimate the seismic strain partition coefficient by comparing the total seismic moment release with that expected from the overall total deformation in the sample observed directly as the mean cumulative strain output from DVC between neighbouring x-ray µCT volumes^21^. The Kostrov strain^39^ was estimated from the seismic moment tensor sum$\:\:{\varDelta\:\varvec{\upepsilon\:}}_{ij}=\frac{1}{2\mu\:\varDelta\:V}\sum\:_{i=1}^{N}{\mathbf{M}}_{ij}$, with moment tensor$\:\:{\mathbf{M}}_{ij}=\sqrt{2{M}_{0}{\mathbf{U}}_{ij}}$, where $\:{\mathbf{U}}_{ij}$ is the unit displacement tensor, $\:\mu\:$ is the shear modulus and Δ*V* is the sample volume. The seismic strain partition coefficient was estimated as $\:\chi\:=\frac{{\gamma\:}_{AE}}{{\gamma\:}_{F}}$, with$\:\:{\gamma\:}_{AE}$ representing the sum of scalar seismic moments multiplied by$\:\:\frac{\sqrt{2}}{2\mu\:\varDelta\:V}$, and$\:{\:\gamma\:}_{F}$ representing the average volumetric and deviatoric strains accumulated throughout the deformation experiment. The seismic strain partition coefficient for events above *m*_*C*_ is estimated to be 3.7% and 0.5% for tests A and B respectively, verifying that deformation is primarily aseismic in both cases, and significantly more aseismic in test B.

### Seismic strain partition coefficient: accounting for undetectable seismicity

One important aspect concerning the strain partition factor estimate was our inability to record small AEs below the relatively high noise level generated by the electrically noisy environment of the Synchrotron facility imaging hutch. This forced omission of smaller events implies that the estimates are a *lower bound* for the seismic strain partition factor. An approach to constrain the *upper bound* for the seismic strain partition factor is to estimate the contribution in the seismic strain partition factor from unrecorded and thereby unaccounted-for low magnitude seismicity involving AE below signal to noise ratios (SNR) and transducer sensitivity limitations. To this end, we considered the theoretical limit of the smallest seismic event by iteratively calculating the seismic strain partition factor, i.e. extrapolating the frequency-magnitude distribution into the range of small events hidden in the noise. The formulae for estimating the contribution of the undetected small amplitude seismicity are presented in the Supporting Information (Text S2). Two further assumptions are used: (a) we use the Brune^45^ source model and link maximum observed crack radius, $\:{r}_{max}$, to the maximum observed magnitude, an approach presented by^44^. $\:{r}_{max}$ was estimated to be 500 μm and 400 μm for test A and B respectively by identifying during visual inspection the longest individual cracks in the vertical plane of each x-ray µCT volume and manually measuring them using the measurement tool in the imaging analysis software FIJI^46^. (b) an assumed smallest crack size used to estimate the smallest event magnitude. By iteratively calculating the source radii and seismic strain partition contribution from smaller magnitude bins (equations S12-S13), we constructed a plot of seismic source radius versus seismic partition coefficient for the two tests (Fig. 7). 100% seismic strain would occur for minimum source radii of 53 nm and 5.4 nm, corresponding to minimum magnitudes of $\:-3.9$ and $\:-5.6$ for the *b*-values obtained for test A and B respectively. These theoretical minimum source radii imply near atomic scale source rupture nucleation for the smallest events, but this is unrealistic^47^ Chap. 1). Hence, it is worth considering independent constraints on the minimum seismic source radius. In the theoretical model of^48^, crack nucleation occurs on pre-existing flaws in the material which act as stress concentrators. Recently it has been demonstrated that a pore-emanating crack model for crack nucleation in porous materials provides a good explanation for the evolution of damage and associated mechanical properties of a range of rock samples with varying porosity, including (with suitable correction for systematic effects) the timing of catastrophic failure^49^. Here we also observe pore-emanating cracks as an important damage mechanism (see section on micro-mechanics of failure above). Given our x-ray resolution limitations, we used the results of^50^, who studied the pore network properties of Clashach sandstone with µCT imaging at a highest resolution of 6 μm. We digitized their frequency pore-size distribution (their Fig. 2a, curve of 6 μm), and weighted the frequency by the pore radius (*r*_*p*_) to account for the fact that larger cracks are more likely to nucleate rupture according to the Griffith theory. We then selected the peak of the distribution as the most likely nucleation crack size for Clashach sandstone. Using the observed peak, the estimated minimum seismic source radius was 9 μm, suggesting an upper bound for the seismic strain partition factors of 8% and 1.3%, with smallest seismic magnitudes of $\:-0.9$ and $\:-1.3\:$for test A and B respectively. The range of seismic strain partition coefficients, 3.7 to 8% for test A, and 0.5 to 1.3% shows how the coefficient is sensitive to the levels of detectability of the smallest event, but remains low.

## Discussion and interpretation

Our results validate our fracture mechanics mixture model for event rate control to high precision ($\:r$ = 99%), and establish its effectiveness in suppressing events of all sizes and hence reducing the maximum magnitude in a finite sample of events. By maintaining event rate in a narrow range, the inferred stress intensity is maintained constant within an even narrower one according to Charles’ law, thereby explaining the remarkable fine control on the stability of deformation in test B. If control is lost, Charles’ law predicts a power-law acceleration in AE event rate with a singularity at the dynamic failure time (e.g^51^). , as observed in test A.

We have also presented further evidence for the inference of constant stress intensity at constant event rate by observing a temporally constant $\:b$-value within the aleatory error limits quoted in Test B. In general there is a negative correlation between *b*-value and stress intensity^52^, so we expect such temporally constant *b-*values for constant stress intensity. Unfortunately there are too few events in test A above threshold to make any clear inferences of temporal trends under constant strain rate loading conditions. However, when there is sufficient AE data in constant strain rate tests, *b*-value commonly reduces with ongoing strain and recovers after dynamic failure^32,44,53–56^. In contrast^16^, who pioneered the use of constant AE event rate feedback in a laboratory setting, found a monotonic decrease of *b*-value to a minimum at the time of fault nucleation in a granite sample, and further recovery of *b*-value during the fault propagation phase, which would imply an increase in stress intensity followed by a relaxation, qualitatively similar to that observed in constrant strain rate loading. However, they also reported a large uncertainty in *b*-value, which may render the trend not statistically significant (the trend is not observable beyond 4 s.d.) We stress the importance of reducing uncertainties in *b*-value estimation in future tests using larger samples with a greater dynamic range of completely reported magnitudes^33^, avoiding the known bias to higher estimated *b*-values in smaller samples^57^, and exploring the effect of sample variability. The slightly higher *b*-value for test B compared to that in Test A implies a lower stress intensity overall for test B, in turn consistent with the stress intensity control hypothesis.

Three further independent observations support the effect of AE rate control on suppressing seismicity, namely the differences between test A and B in (a) maximum magnitude, (b) the total number of events, and (c) the seismic strain partition coefficient. As shown in Fig. 2c, d, the AE rate control leads to suppression of maximum magnitude by almost one unit, and similarly leads to a smaller number of events of all sizes. This is in turn consistent with the lower seismic efficiency for test B by almost an order of magnitude compared with constant strain rate control. Our inferences on the seismic moment budget are based on calibration of the maximum crack length involving the Brune model for the seismic source. Example spectra for the two tests are shown in Supplementary Information (Figure S4). The results show that the largest ten AE events from both experiments had similar corner frequencies. The independent observations of similar crack sizes and corner frequencies implies that rupture velocities for individual dynamic events were similar. Together with the higher inferred seismic moments for test A, this implies larger stress drops for these AE sources in test A, based on the Brune source model (see eq. S1). While small differences in stiffness (Young’s modulus) were observed between the two samples in the common elastic stage of loading, the difference in this intrinsic stiffness is not large enough on its own to account for the differences in maximum magnitude (1.4 for test A cf. 0.9 for test B), and so the differences in stress drop must be due to the loading protocol, with quasi-static loading promoting a lower stress drop during seismic events.

Several field studies address the geologic evolution of faults, showing that they grow in a scale-invariant or self-similar way, albeit with a large scatter about the mean trend (e.g^58,59^). The scatter is most likely due to heterogeneous fault properties, including roughness, and consequently a heterogeneous stress field which in turn could affect stress drop during fault rupture (e.g. refer to^44^ and references therein). In the laboratory, where finite size sample effects also play a role, Blanke et al.^60^ observed from their stick-slip experiments on fractured vs. smooth granite that: (i) there is a systematic scaling of stress drop with AE size, (ii) the scaling is governed by fault roughness. Their findings suggest that fault zone heterogeneity (i.e. roughness and grain size distribution) may also affect the stress drop of AEs, which may apply to some extent to our experiments. In the same vein, we expect lithology to impact stress drop and seismic energy release, however, in the absence of a comparative study, we don’t know yet how stress drop, and its scaling with magnitude, depends on rock type, or whether these parameters explain the inferred variability of stress drop, or a systematic effect that is significant within this scatter, or both.

Our results suggest there is a critical AE event rate threshold to stabilize fracture growth. An event rate of 2 events/sec was observed during test A for a minute or so before the event rate rapidly accelerated immediately before peak stress. Consequently, a lower threshold of 1 event/sec was chosen as a maximum event rate for stable sub-critical cracking before shear zone localisation. This was sufficient to suppress run-away uncontrollable acceleration compared to higher AE event rate feedback settings. For feedback control at 1 AE/s, the actual incremental AE rate fluctuated between 0 and 2 events/s. In future experiments on larger or different sample types, similar preliminary tests would be required to optimize the protocol, in addition to improved signal to noise through pre-amp gain optimization. As shown by^61^, aseismic slip could also trigger induced seismicity by propagating stress in fractures and faults that are critically stressed. While AE rate control may succeed in maintaining a system in a sub-critical state, it is still not known whether seismic monitoring controls can be effectively used to prevent catastrophic failure in systems that are already critically stressed. Further laboratory testing of critically stressed systems is scheduled to address this.

Induced seismicity is often associated with reactivation of preexisting faults or fractures (e.g^62^). While our experiments describe failure behaviour of intact rock., our findings reflect some of the behaviour of shearing along pre-existing fractures, in the following instances: (a) once the initial damage is localized within the intact rock, such as the observed en echelon fractures presented above, that in itself is the plane of weakness along which shearing consequently occurs, with potentially similar damage evolution to a pre-existing fracture system; (b) if pre-existing faults have not slipped for long periods, and healed under physical and chemical processes, they may behave in similar ways to intact rock breaking; (c) loading of a pre-existing fault may still create new secondary faults within surrounding intact rock matrix, if existing fault orientation is not favourable^63,64^); (d) loading of a pre-existing fault that is not healed can still involve breaking of asperities in the case of rough faults.

The experiments were performed under water-saturated but drained conditions, a common protocol for experimental deformation of porous rock. Given that our samples were high porosity, and probably high permeability (as suggested by^65^ who analyzed the response of Clashach sandstone’s permeability to loading), we expect fully drained conditions throughout both tests, apart from during rupture in test A. However, to assess the validity of the drainage conditions would require the experimental apparatus to include an upstream fluid port for upstream flow rate / passive pressure to allow measurement of fluid gradients. In the absence of these measurements, we cannot validate the drainage conditions. Alternatively, we propose as a future study the modelling of the permeability evolution from the x-ray images, and consequent estimation of fluid flow.

In terms of potential applications to fluid-pressure induced seismicity, we acknowledge that we did not drive the test by varying the fluid pressure: our protocol increased the effective differential stress $\:{\sigma\:}_{e,1}={\sigma\:}_{1}-{P}_{P}$ under static stress intensity conditions at constant fluid pressure, rather than the other way round. Our experiments for constant strain/AE rate loading, leading to dynamic/quasi-static failure processes, respectively, suggest that low amplitude load oscillation to maintain a constant AE rate reduces the effective strain rate, promotes aseismic behaviour and reduces maximum magnitudes. Ji et al.^66^ show that cyclic injection can reduce the fluid over-pressure ratio and the max seismic moment as a result and Ji et al.^67^ show that cyclic injection promotes slow and stable slip and is therefore effective in mitigating injection-induced seismic hazard. On the other hand, Ji et al.^68^ show that high-rate fluid injection constitutes dynamic loading and that faster injection rates may promote transition from aseismic to seismic slip, leading to higher magnitude events and increased injection-induced seismic hazard. This suggests that injection rate and loading rate can have qualitatively similar effects, albeit the injection rate effect would be expected to have a delay due to finite permeability of the medium.

The primary difference in fluid injection under constant tectonic stress would be the introduction of a delay in remote feedback due to the effects of fluid pressure diffusion. This delay would be especially notable in field scenarios where diffusion times are extended. However, it mirrors the issue seen in the existing ‘traffic light’ system, which also experiences a comparable delay and has led to numerous consequential ‘trailing events’ following shut-in. In EGS operations, Kwiatek^8^ showed that by iteratively reducing injection rates, and extending waiting periods between pumping phases, more dynamic rupture propagation can be avoided, consequently maintaining magnitudes below permitted thresholds. This had the effect of maintaining a relatively constant *b*-value and keeping the event rate below certain threshold – mirroring the results of our study despite the change in boundary conditions and scale. We acknowledge that implementation of other physics-based approaches has also proven to be effective in induced seismic risk management. For example, Zang et al.^9^ showed that using cyclic pressurization and pulse pumping to induce fatigue hydraulic fracturing systematically reduced induced seismic magnitudes due to reduced breakdown pressure.

Our findings explain how controlling seismic event rates can reduce the likelihood of extreme events, consistent with observations of its use in coal mining^69^. They also provide a rationale for extending the application of event rate control in fluid-injection scenarios with the caveats stated above. Further analysis is required to address the differences in the physics between crack growth vs. reactivation of existing faults under fluid injection, and the relationship of injection control parameters to induced seismicity rates.

Finally, we recognize that repeat tests, which were not performed due to beamtime constraints, could substantiate some of the findings of this study. However, since both tests were conducted under the same conditions until the AE feedback control took over, we have a benchmark for a degree of repeatability (albeit with a small but systematic difference in stiffness between the tests) up to the yield point, as evidenced by the average local strain evolution shown in Fig. 3. However, we recognize it is a weakness in the current study, and during future beamtimes, we will address the issue of repeat tests.

## Conclusions

Our main findings validate the hypotheses set up in the introduction. First, the deformation mechanisms observed in the x-ray images for crack development under constant strain rate and constant event rate are different, notably with a smaller proportion of damaged rock, less fragmentation and gouge, a delayed onset of localisation and a narrower shear band in the constant AE rate test. Second, the stress history for the constant AE rate control validates our new physical model for event rate control based on the empirically-observed relationship between stress intensity and AE rate and an evolving mixture of hardening and softening mechanisms expected in a compressive stress field and observed in the evolving microstructure. In particular the observed differential stress history under constant AE rate loading is independently consistent with the theory with a high regression coefficient (99%), explaining how failure is stabilized in the post-peak regime by maintaining a constant, sub-critical, stress intensity or crack extension force. The inference of constant stress intensity is independently consistent with a temporally-constant AE *b*-value. Third, the number of events of all sizes is lower under AE feedback control, in turn reducing the likelihood of extreme events. As a consequence, the observed maximum magnitude is just under one unit lower, and the proportion of seismic to total strain is almost an order of magnitude lower in the constant AE event rate test compared with the constant strain rate test. Our results explain the empirical observation at field scale that keeping event rate below a given level can result in an effective control on the likelihood of extreme events in coal mining, and suggest that adding event rate control to that of maximum recorded magnitude may be more effective than maximum event magnitude threshold ‘traffic light’ systems alone. The effectiveness of event rate control in the presence of pre-existing fractures, proximity to criticality and fluid-induced fault re-activation requires further research, in laboratory and field scale testing.

## Electronic supplementary material

Below is the link to the electronic supplementary material.

Supplementary Material 1

## Data Availability

The raw acoustic waveforms and raw mechanical data, along with the reconstructed 3D X-ray μCT volumes, generated in this study are available at the NERC EDS National Geoscience Data Centre repository under accession code: https://webapps.bgs.ac.uk/services/ngdc/accessions/index.html#item173296, with direct access to the dataset available at the NERC STFC Centre for Environmental Data Analysis third party holdings: https://data.ceda.ac.uk/ngdc/NE_R001693_1. These data are available under Open Government Licence (OGL). When using the dataset held in the repository, please cite Cartwright-Taylor et al. (2022). The raw X-ray μCT radiograph files are very large and are stored at the Diamond Light Source. They are available on request from the authors. All other processed data supporting our conclusions can be found in the main manuscript and in the Supplementary Information.
