# Supplementary material for: Loading of a porous rock with constant micro-seismic event rate suppresses seismicity and promotes subcritical failure
Source: Sci Rep. 2025 Jun 3;15:19351. doi: 10.1038/s41598-025-03105-5 (PMC12130455; doi:10.1038/s41598-025-03105-5)

**Supplementary Information**

**Contents of this file**

Text S1 to S2

Figures S1 to S4

**Additional Supporting Information**

Table S1

**Text S1**. Implication of a *b*-value over 1.5

The Gutenberg-Richter (G-R) law defines a log-linear relationship between the frequency of occurrence of earthquakes and their magnitude:

$logF\left( m \right)=a-bm$ (S1)

where *F(m)* is the incremental frequency of occurrence of earthquakes with magnitudes in the range $m\pm\frac{\delta m}{2}$ , with $\delta m$ corresponding to the bin width and $a$*, b* constants.

The implication of a *b*-value over 1.5 is explained as follows. One can express $F$ as a function of *m_min_* and *b*, where *m_min_* should be equal to or above the threshold magnitude for complete detection of events *m_C_*, as follows:

$\Delta\left( logF \right)=logF-\log F\left( m_{min} \right)=\left( m-m_{min} \right)b \Rightarrow F=F(m_{\min}){10}^{-b(m-m_{min})}$ (S2)

The magnitude (*m*) is a logarithmic measure of seismic moment (*M*):

$logM=cm+d$ (S3)

where it is commonly assumed that $c=\frac{3}{2}$ [70].The total moment in each bin is:

$FxM=F\left( m_{min} \right){10}^{-b\left( m-m_{min} \right)}{10}^{cm+d}=F\left( m_{min} \right){10}^{-m\left( c-b \right)+bm_{min}+d}$ (S4)

In the case of *b = 1.5*, *c-b = 0*, *FM = constant*, which suggests that each bin above *m_min_* contributes equally to the moment sum. If *b > 1.5*, such as in our dataset, *FxM* is bigger for smaller events, which translates to smallest events contributing most to seismic strain – and conversely, if *b < 1.5*, *FM* is bigger for larger events (largest events contribute most).

**Text S2**. Accounting for undetectable seismicity in the seismic strain partition coefficient

Using the Brune [43] model, we estimate the seismic moment as a function of the square of the seismic source radius:

$M=K_{1}\Delta\sigma r^{2}$ (S5)

where *r* is the seismic source radius, *K_1_* is a constant, and *Δσ* is the stress drop.

Using (9), the ratio of maximum to minimum magnitude can be expressed as:

$\frac{M_{max}}{M_{min}}=\frac{{10}^{cm_{max}+d}}{{10}^{cm_{min}+d}}=\frac{r_{max}^{2}}{r_{min}^{2}}$ (S6)

$\Rightarrow r_{min}=r_{max}{10}^{\frac{1}{2}c(m_{min}-m_{max})}$ (S7)

Here the term $K_{1}\Delta\sigma$ cancels on division, so the calibration is independent of the absolute stress drop. Next, we look at the seismic moment (*M*) sum over *n* individual entries *i*, which is related to the sum over *q* magnitude (*m*) bins:

$\sum_{i=1}^{n} M_{i}=\sum_{j=1}^{q} F\left( m_{j} \right)M\left( m_{j} \right)=\sum_{j=1}^{q} {10}^{1-bm_{j}+cm_{j}+d}=K_{2}\sum_{j=1}^{q} {10}^{(c-b)m_{j}}$ (S8)

where *K_2_* is a constant.

We now try two different threshold magnitudes, one being the magnitude of complete reporting (*m_C_*), with *n_C_* events and *q_c_* bins, and one assuming the magnitude due to the smallest crack possible, with *n_max_* events and *q_max_* bins. By proportion:

$\frac{\sum_{i=1}^{n_{max}} M_{i}}{\sum_{i=1}^{n_{c}} M_{i}}=\frac{\sum_{j=1}^{q_{max}} {10}^{(c-b)m_{j}}}{\sum_{j=1}^{q_{c}} {10}^{(c-b)m_{j}}}$ (S9)

The total strain measured from the observed deformation is *ε_T_*. The seismic strain *ε_S_* is based on Kostrov (1974), and defined as:

$\varepsilon_{S}=K_{3}\sum_{i=1}^{n} M_{i}$ (S10)

with *K_3_* constant, while the seismic strain partition factor $\chi=\frac{\varepsilon_{S}}{\varepsilon_{T}}$ for the two thresholds are similarly related by direct proportion:

$\frac{\chi_{q_{max}}}{\chi_{q_{C}}}=\frac{\sum_{j=1}^{q_{max}} {10}^{(c-b)m_{j}}}{\sum_{j=1}^{q_{C}} {10}^{(c-b)m_{j}}}$ (S11)

We further compute the reduction in radius implicit in reduction of magnitudes for neighbouring bins of thickness *Δm = 0.1* as:

$r_{j-1}=r_{j}{10}^{-\frac{1}{2} c 0.1}=r_{j}{10}^{-\frac{1}{2} 1.5 0.1}=r_{j}{10}^{-0.075}$ (S12)

and extrapolate downwards the radii until we reach an acceptable lower limit for the seismic source radius. In addition, we compute the increase in strain partition factor from smaller magnitude AEs in neighbouring bins from eq. (S11) as:

$\chi_{j}=\chi_{j-1}{10}^{(c-b)(m_{j}-m_{j-1})}=\chi_{j-1}{10}^{(1.5-b)(0.1)}$ (S13)


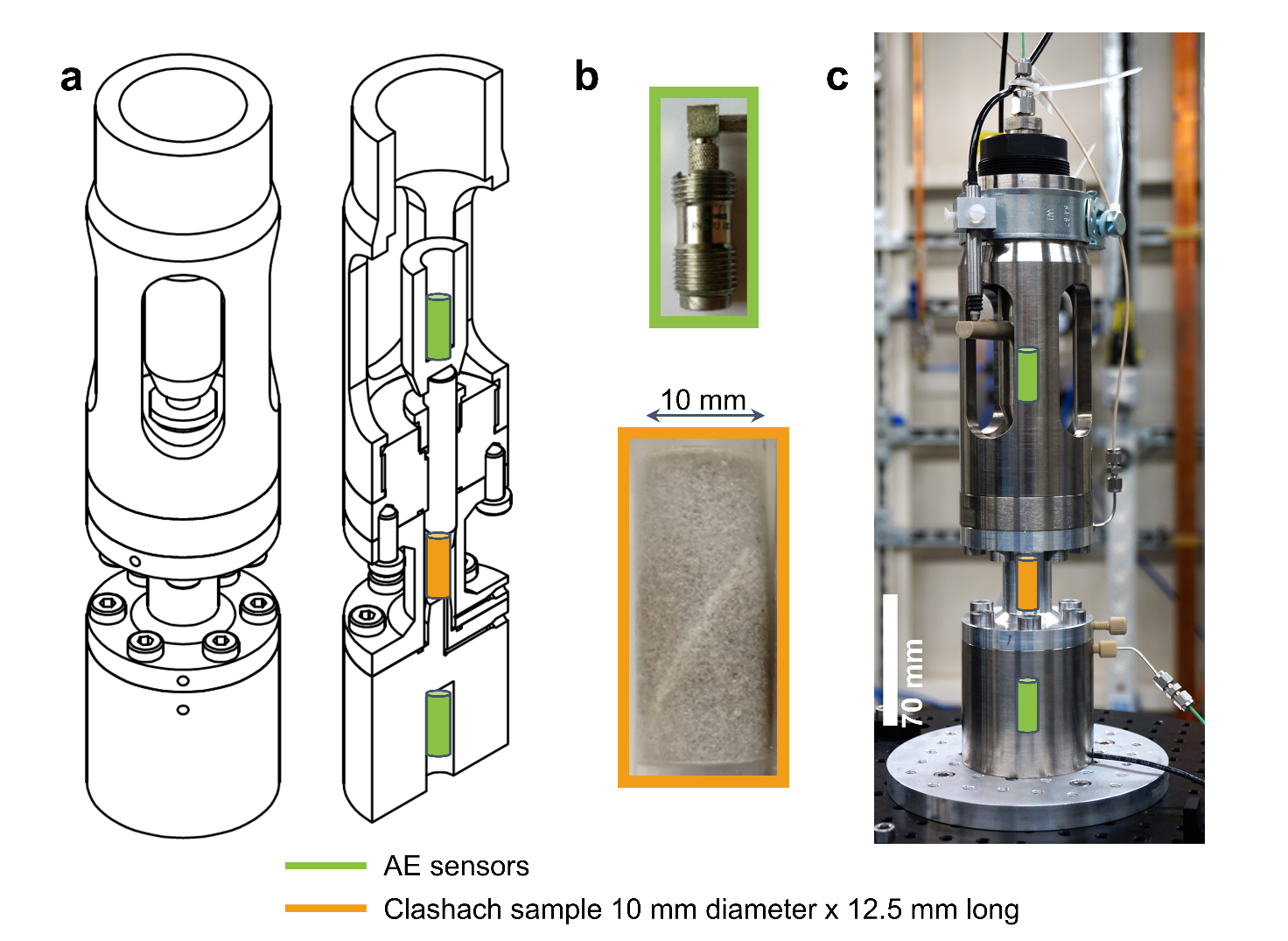


Figure S1. Stór Mjölnir – our x-ray transparent rock deformation cell. a Schematic and c photograph of our x-ray transparent rock deformation cell, Stór Mjölnir, with acoustic emissions (AE) monitoring. b Clashach sandstone sample (bottom; orange box) sits in the pressure vessel, and two piezoelectric transducers (top; green box) sit at either end of each piston, as shown in a and c. Axial displacement was measured outside the pressure vessel with a linear variable displacement transducer (LVDT).


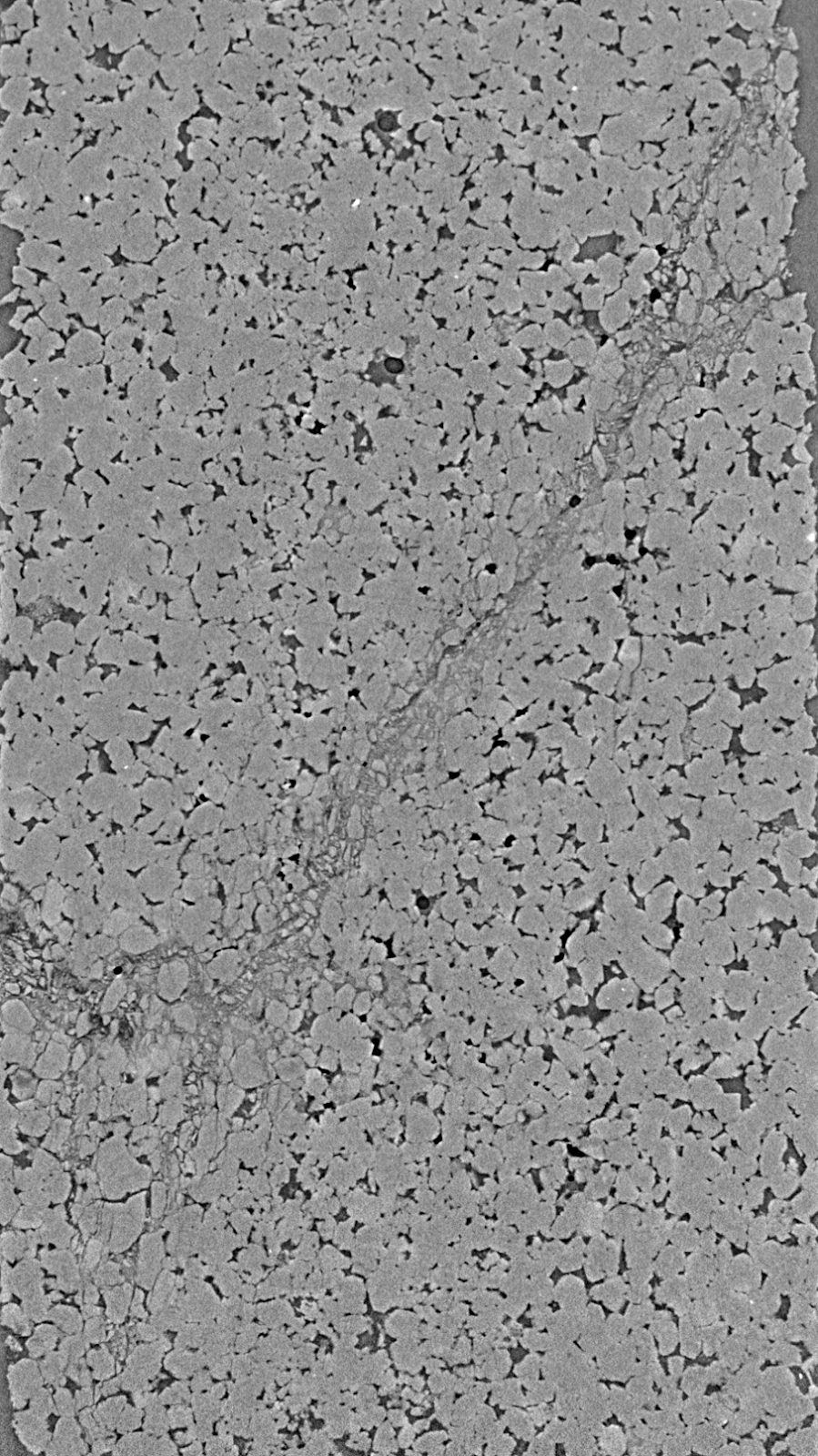


**Figure S2.** X-ray cross-section of failed sample for test A (diameter = 10mm).


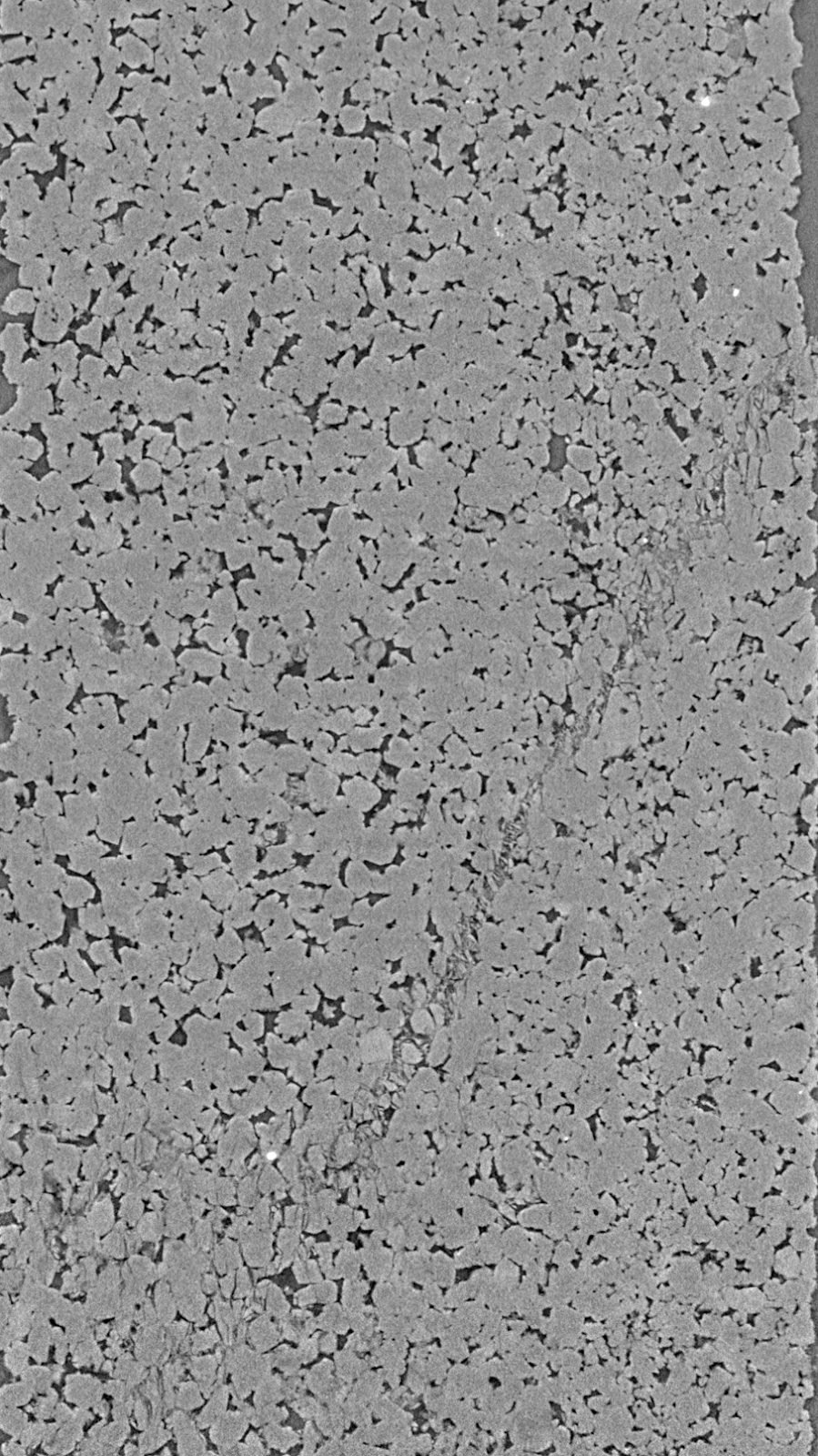


**Figure S3.** X-ray cross-section of failed sample for test B (diameter = 10mm).


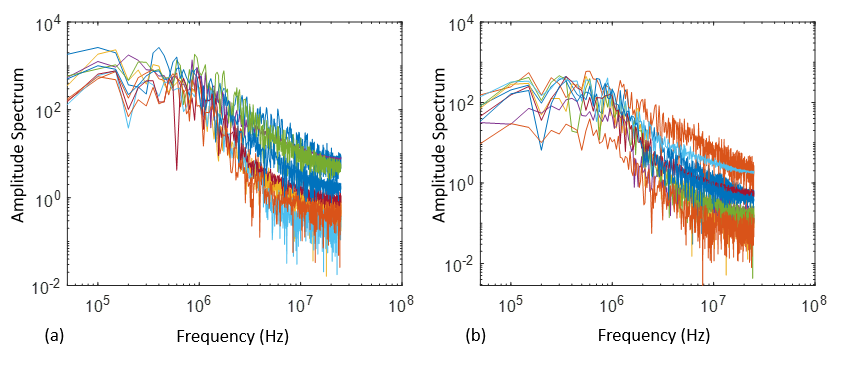


**Figure S4. Amplitude spectra of ten largest events from AE dataset of test A (a) and B (b). Corner frequencies are fairly similar for all events.**

**Additional Supporting Information**

**Table S1.** Temporal variation in b-values for Test A (top table) and B (bottom table).


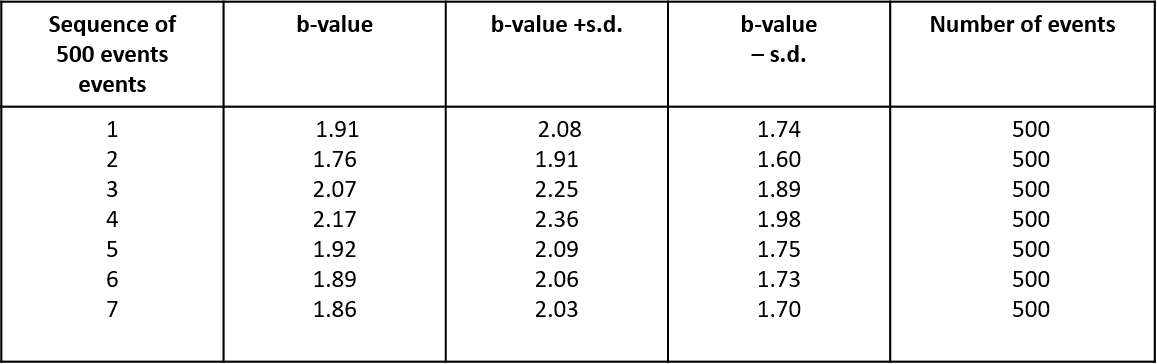

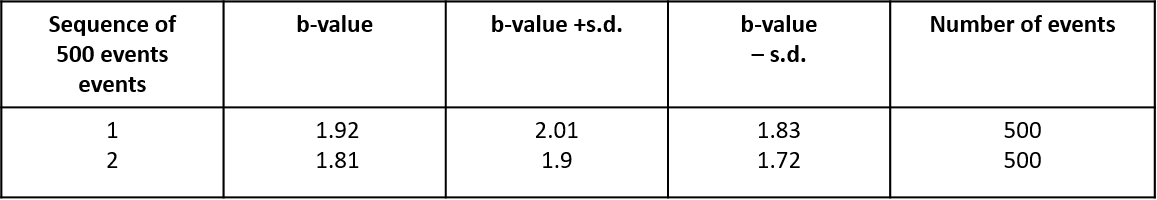

Supplement: Supplementary file 1 — Supplementary Material 1 [file 41598_2025_3105_MOESM1_ESM.docx]
